# An optimised method for quantifying glenoid orientation

**DOI:** 10.4103/0973-6042.41407

**Published:** 2008

**Authors:** Hippolite O. Amadi, Sughran Banerjee, Ulrich N. Hansen, Andrew L. Wallace, Anthony M. J. Bull

**Affiliations:** Department of Bioengineering, Imperial College London, South Kensington Campus, London SW7 2AZ, UK; 1 Shoulder Unit, Hospital of St John and St Elizabeth, 60 Grove End Road, London NW8 9NH, UK

**Keywords:** Version, inclination, morphology

## Abstract

A robust quantification method is essential for inter-subject glenoid comparison and planning of total shoulder arthroplasty. This study compared various scapular and glenoid axes with each other in order to optimally define the most appropriate method of quantifying glenoid version and inclination.

Six glenoid and eight scapular axes were defined and quantified from identifiable landmarks of twenty-one scapular image scans. Pathology independency and insensitivity of each axis to inter-subject morphological variation within its region was tested. Glenoid version and inclination were calculated using the best axes from the two regions.

The best glenoid axis was the normal to a least-square plane fit on the glenoid rim, directed approximately medio-laterally. The best scapular axis was the normal to a plane formed by the spine root and lateral border ridge. Glenoid inclination was 15.7° ± 5.1° superiorly and version was 4.9° ± 6.1°, retroversion.

The choice of axes in the present technique makes it insensitive to pathology and scapular morphological variabilities. Its application would effectively improve inter-subject glenoid version comparison, surgical planning and design of prostheses for shoulder arthroplasty.

## INTRODUCTION

Effective surgical planning for total shoulder arthroplasty requires a clear understanding of a patient's glenoid version and inclination,[1–6] Quantification of these parameters even in the presence of osseous pathology requires a robust and reproducible technique.[7] Several methods have been proposed for *in vivo* quantification of glenoid version; from the use of conventional roentgenograms to axial-tomographic scans.[4,8,9] Computed tomographic (CT) methods are more reproducible and reliable compared to conventional X-ray methods.[10,11]

**Figure 1:**
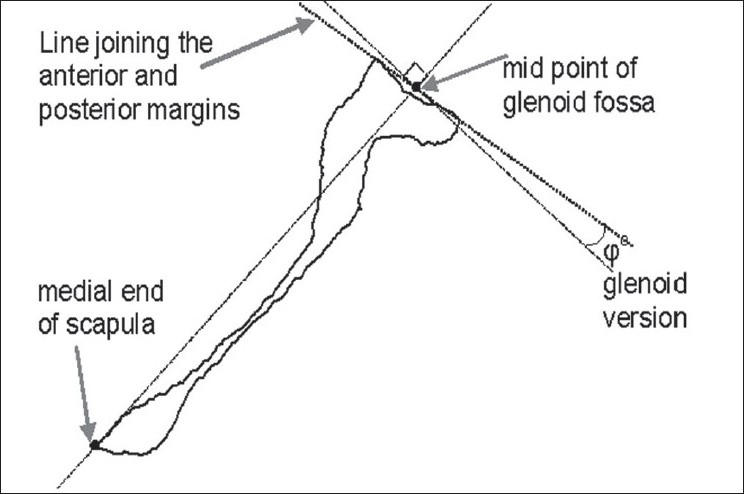
Mid glenoid section illustrating version angle due to Friedman *et al*. 1992

Friedman *et al*.,[8] used a method that requires three landmarks to define glenoid version. They used CT scans in the axial plane from the acromion to the inferior border of the glenoid. Glenoid version was measured on the slice corresponding approximately to the mid-glenoid level [Figure 1]. Although an improvement on conventional X-ray methods[10] there remain limitations to this technique in that the results are scanning-orientation dependent;[3,9,12] it is essential that the glenoid surface is perpendicular to the plane of the CT slice. An improvement is to use ultrasound to define the perpendicular to the glenoid face.[12] It is known that the glenoid face is twisted in a superior-inferior direction[3,4] and therefore the use of two points from a subjective mid-glenoid slice will be susceptible to inherent errors. Others have used methods with either surface scanning[4] or direct physical measurements[6] of *ex vivo* scapulae. These methods suffer from scanning orientation dependency that is set by eye,[4] or use of only two points to define an angle.[6]

In another study, Couteau *et al*.[9] carried out a 3-D morphological and mechanical analysis of twelve shoulders using CT scans. In their method, the points defining the glenoid articular surface were extracted and their centroid calculated. A least-square (LS) plane was mathematically fitted on the extracted points and a normal unit vector to this quantified. This represented the glenoid axis. A mid-transverse section of the glenoid was defined as the axial slice corresponding to the location of the centroid. The central axis of inertia of this slice was quantified to represent the scapular axis. The version angle was finally calculated as the angle between the two representative axes.

Fundamentally, glenoid quantification can be seen as the measurement of the glenoid plane orientation relative to the scapular plane. All the earlier techniques achieved this by applying two axes, one each to represent the planes. Most of these techniques rely on three or fewer landmark points that are susceptible to failures in the presence of pathologies. Again, it is known that inter-subject variability in the morphology of the scapula exists which none of these techniques addressed.[13,14] Therefore, comparison of glenoid quantification between individuals using these techniques might not be reliably accurate. A more reliable technique could be developed based on axes that address the known limitations, having minimal inter-subject variability as well as being pathology-independent.

**The aim of this work was to:**

1. Compute the axes of the glenoid and scapula as defined in the literature as well as other axes defined here from clearly identifiable landmarks,
2. To use weighting criteria to compute the best axes that are least susceptible to morphometric variability to define glenoid version and inclination.

## MATERIALS AND METHODS

Three-dimensional image datasets from standard shoulder scans were assessed for obvious osseous pathology and twenty-one of them selected. This comprised seventeen CT image scans and four cryosectional image datasets. Sixteen of the specimens were left shoulders, mean age was 60 years, range (57 years to 79 years). Nine of the image scans were of 1.00 mm slice thickness, six (1.50 mm), four (1.40 mm) and two (1.25 mm).

Features or regions of interest within the field of view of any standard shoulder or chest scan were defined. This includes regions within the scapular distal half and the supraglenoid tubercle. AMIRA image processing software (Mercury Computer Systems Inc, Chelmsford, MA, USA) was applied to segment and extract the three-dimensional locations describing each feature of interest.

**Figure 2:**
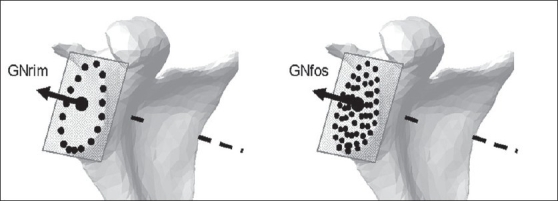
Normal unit vectors to the glenoid

**Figure 3:**
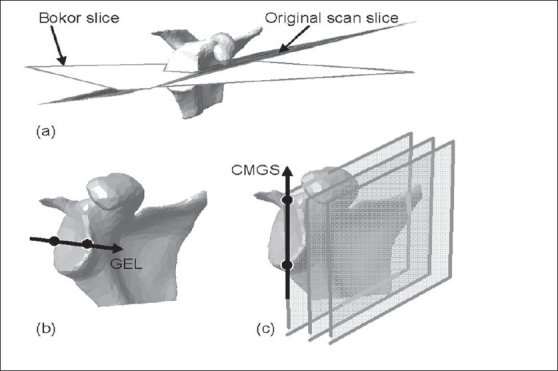
(a) Re-slicing to conform to Bokor et al's proposal (b) Glenoid equatorial line and (c) Coronal mid-glenoid axis

Least-square basic geometric shapes such as an ellipse, plane, line or triangle were numerically fitted on a given set of points to quantify axes on each specimen. These include those normally applied by classical techniques for glenoid quantification and some novel ones. The specific axes that were defined are described below:

- Glenoid rim normal (GNrim): This is the normal unit vector to the best-fit plane over the rim of the glenoid. The outline of the glenoid rim was segmented, reconstructed and applied to mathematically quantify the least square plane-fit over the points and the normal unit vector to it [Figure 2].
- Glenoid fossa normal (GNfos): The normal unit vector to the best-fit plane over the glenoid fossa.[9] The entire glenoid fossa was segmented, reconstructed and applied to quantify the plane and its normal [Figure 2].
- Glenoid equatorial line (GEL): A line joining the anterior and posterior margins of the mid-glenoid slice.[4,8,10] This is the axial slice midway along the glenoid height [Figure 3].
- Coronal mid-glenoid superior axis (CMGS): A line joining the inferior and superior margins of the mid-glenoid slice from the coronal frames of an image scan [Figure 3]. This is the coronal slice midway along the glenoid width.
- Bokor glenoid equatorial line (BGEL): This is a GEL based on the proposals of Bokor *et al*.,[12] that scan orientation should be such that the glenoid surface is perpendicular to the plane of the CT axial cut [Figure 3]. This was achieved using image processing software.
- Glenoid superior axis (GSA): A line directed superiorly from the most inferior aspect of the glenoid to the biceps tendon insertion.[5,6,15]

**Figure 4:**
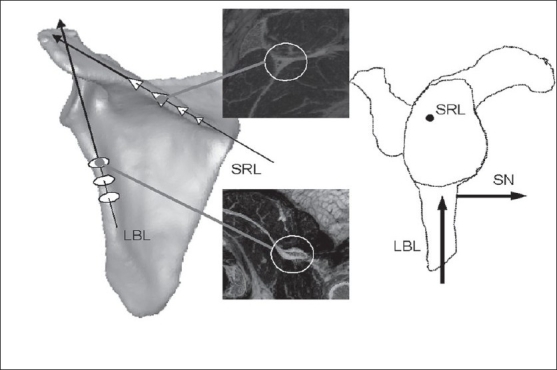
Normal unit vector to the scapular body and its parent vectors

For the scapula, the axes were:

- Lateral border line (LBL): The best-fit inferior-superior line along the ridge of the scapular lateral border [Figure 4].
- Spine root line (SRL): The best-fit long-axis along the root of the scapular spine [Figure 4].
- Scapular normal (SN): The cross-product (unit vector) between LBL and SRL [Figure 4]. This is directed anteriorly.
- Scapular transverse axis (STA): A line drawn from the mid point of the glenoid fossa to the medial edge on the mid glenoid transverse slice[8] [Figure 1].
- V. Bokor scapular transverse axis (BSTA) as proposed by Bokor *et al*.[12]
- Second Moment of Area transverse Axis (SMATA): The medio-laterally directed principal axis of the second moment of area quantified on the closest axial slice to the centroid of the glenoid fossa.
- Wong scapular transverse axis (WSTA): This is a line joining the spinoglenoid notch and the spine/medial border intersection.[5]
- Churchill scapular transverse axis (CSTA): This is a line joining the centre of the glenoid fossa and the spine/medial border intersection.[6]

The corporate morphology of the glenoid or scapula was characterized by these axes that were defined from landmarks. It is therefore essential to identify a glenoid axis that integrates the variations in the remaining axes in its make-up. Such an axis would therefore be relatively insensitive to changes in glenoid morphology represented by inter-subject variations in the remaining axes. For the scapular body also, the best axis capable of reflecting this quality was required.

The angles between all the glenoid axes were calculated in all the specimens. The means and standard deviations (SD) for these were quantified. A relatively insensitive axis would result in a smaller sum total of its SDs from the specimens compared to the rest of the axes. The insensitivity index of an axis was defined as the sum of its SDs from the 21 specimens. All the insensitivity indices were normalized relative to the smallest index which assumed a weighting value of 1. ‘Relative Insensitivity’ of the rest of the axes was thus quantified. These were also done for the scapular axes. In addition to high insensitivity, the final criterion for the selection of the best axes was based on pathology-independency. An axis of which quantification was based on two or three points only had a risk of pathological failure if any of the quantification landmarks was associated with any regular osseous pathology. Such an axis was assigned a weighting of 1, otherwise this was 0. Optimal glenoid version was defined as a measure of the angle between the best glenoid axis and that of the scapula on the approximate transverse plane. Optimal glenoid inclination was defined as a measure of the angle between the best glenoid and scapular axes on the approximate coronal plane. Four different classical techniques were also applied to quantify version of each specimen. These were: (I) Friedman *et al*.,[8] angle between GEL and STA; (II) Bokor *et al*.,[12] angle between BGEL and BSTA; (III) Couteau *et al*.,[9] (modified) angle between GNfos and SMATA; (IV) Churchill *et al*.,[6] angle between BGEL and CSTA. Glenoid inclination was quantified using two other methods: (I) Wong *et al.,*[5] method as the included angle between WSTA and GSA; (II) Churchill *et al*.,[6] method as the angle between CSTA and GSA. The correlation coefficients between these and the optimal methods were also calculated.

## RESULTS

The most insensitive axis of the glenoid is the normal to a LS plane fit on the glenoid rim (GNrim) while that of the scapula is the normal to the plane formed by LBL and SRL (SN). These have Relative Insensitivity of 1.00 respectively [Tables 1 and 2]. Quantification of these involved multitudes of points over their landmarks. Optimal glenoid version is therefore a measure of the angle between GNrim and SN. This produced a mean value of 4.9 ± 6.1°, retroversion; range: -16.4° to 10.7°. Mean glenoid version using Friedman *et al*.[8] technique was 12.2° ± 8.4°, retroversion; range: -30.6° to 0°; having correlation coefficient of 0.08 with the optimal method. Bokor *et al*.,[12] technique on the same specimens produced mean glenoid version of 3.5° ± 4.8°, anteversion; range: -4.5° to 14.5°; and correlation coefficient of 0.26 with the optimal method. By Cauteau *et al*.,[9] parallel technique, this was 15.8° ± 38.2°, anteversion and correlation coefficient of 0.12. Churchill *et al*.,[6] method produced 3.3° ± 4.6°, anteversion; range: -4.6° to 13.1°; and correlation coefficient of 0.23. The CSTA and WSTA with equal Relative Insensitivity of 1.02 are the most insensitive scapular axes on the approximate coronal plane. These were followed closely by SRL (relative insensitivity, 1.03). By pathology-independency criterion, the SRL was quantified with numerous points as against the two-point and pathology-dependent CSTA and WSTA. This was therefore chosen as the best. This combines with the glenoid's GNrim to produce an ‘optimal’ mean glenoid inclination of 15.7° ± 5.1°, superiorly; range: -7° to 27.4°. Wong *et al*.,[5] method quantified a mean inclination of 0.9° ± 4.3°, superiorly; range: -7.1° to 11.2°. Churchill *et al*.,[6] method produced 5.2° ± 3°, superiorly; range: 0.8° to 11.5°.

**Table 1:** Relative insensitivity and pathology dependency in glenoid axes

Axes	RI	PD	Direction	No of points involved
GNrim (Glenoid rim normal)	1.00	0	medio-lateral	Thousands
GNfos (Glenoid fossa normal)	1.67	0	medio-lateral	Thousands
GSA (Novotny's line)	1.23	1	infero-superior	2
BGEL (Bokor's line)	1.33	1	antero-posterior	2
GEL (Friedman's line)	1.50	1	antero-posterior	2
CMGS (Mid-glenoid i-s line)	2.44	1	infero-superior	2

RI - Relative insensitivity, PD - Pathology dependency

**Table 2:** Relative insensitivity and pathology dependency in scapular axes

Axes	RI	PD	Direction	No of points involved
SN (SRL-LBL plane normal)	1.00	0	antero-posterior	Thousands
SRL (Spine Root line)	1.03	0	medio-lateral	Thousands
WSTA (Wong's line)	1.02	0	medio-lateral	2
LBL (Lateral Border Line)	2.00	0	infero-superior	Thousands
CSTA (Churchill's line)	1.02	1	medio-lateral	2
BSTA (Bokor's line)	1.07	1	medio-lateral	3
SMATA (2nd Moment Area)	2.52	0	medio-lateral	Thousands
STA (Friedman's line)	1.89	1	medio-lateral	3

RI - Relative insensitivity, PD - Pathology dependency

## DISCUSSION

The classical methods of glenoid version quantification have been associated with various limitations such as scanning orientation dependency.[3,4,9,12] More recent studies have proposed other methods that addressed the orientation factor. However, these are also flawed for being sonographer-dependent in ensuring preferred scanning orientation. The technique proposed in the present study was based on thousands of vectors to form the SRL, SN and GNrim axes. GNrim integrates the corporate morphology of the glenoid rim rather than two points only compared to other methods.[6,8,12] This would therefore remain stable irrespective of the scanning orientation unlike the techniques of Friedman *et al*.[8] and Monk *et al*.[4] and hence avoids the subjective opinion of the sonographer. The rim of the glenoid has been reported to be superoinferiorly twisted and might have the presence of osseous pathology.[3,7,9] The fitting of LS plane over the glenoid face using over two thousand points across the glenoid rim constitutes a better approximation of glenoid definition irrespective of the presence of the aforementioned complications. The SN axis integrates most of scapular morphology represented in over 5000 points from its parent axes (SRL and LBL). This is therefore a better representation of the scapular body compared to only two points applied by the classical methods. None of the earlier techniques produced a good correlation with the present technique because of its unique approach. This used an ‘anterior-posterior’ axis for the scapula instead of ‘medio-lateral’ axis applied by others.

Glenoid inclination has not been as extensively discussed in the literature as the version. This might suggest that the parameter is not seen to be so important during shoulder arthroplasty. However, it is known that a more upward-facing glenoid increases the risk of superior humeral head migration, possibly associated with the genesis of rotator cuff disease.[5] Similar to the classical method of version calculation, inclination is based on defining a line joining two points only on the glenoid rim.[5,6] The second line has been differently defined in the literature. Churchill *et al*.,[6] line joined the spine-medial border intersection to the glenoid fossa centre while Wong *et al*.,[5] joined this to the spinoglenoid notch. The present study however, has demonstrated glenoid inclination based on a multipoint approach, using GNrim and SRL.

Quantifications based on the present proposals are easily realized using any standard shoulder or chest scans and do not require any special radiological scan of the patient. The derivation and application of subject-invariant axes in this study would allow a more accurate inter-subject comparison of glenoid quantification. This could allow better design of prostheses and ensure a more effective surfacing of the glenoid during total shoulder arthroplasty. The present technique's sensitivity to numerically describing version and inclination and its insensitivity to scanning orientation suggest that this has the potential to be a clinical tool in assessing glenohumeral function. As a numerical technique, this can be automated and considerable time saved for the quantification of these parameters. Further studies will have to be conducted to relate these parameters of version and inclination to clinical outcome.
